# Identification of novel *SHANK2* variants in two Chinese families via exome and RNA sequencing

**DOI:** 10.3389/fnins.2023.1275421

**Published:** 2023-11-24

**Authors:** Yong Wu, Wenzhou Li, Bo Tan, Sanchuan Luo

**Affiliations:** ^1^Medical Research Institute, Shenzhen Baoan Women’s and Children’s Hospital, Shenzhen, China; ^2^Department of Gynecology and Obstetrics, The Second Affiliated Hospital of Chongqing Medical University, Chongqing, China

**Keywords:** *SHANK2*, neurodevelopmental disorder, whole-exome sequencing, RNA sequencing, cohort analysis

## Abstract

**Background:**

*SHANK2* encodes a postsynaptic scaffolding protein involved in synapse formation, stabilization and homeostasis. Variations or microdeletions in the *SHANK2* gene have been linked to a variety of neurodevelopmental disorders, including autism spectrum disorders (ASD) and mild to moderate intellectual disability (ID) in human. However, the number of reported cases with *SHANK2* defects remains limited, with only 14 unrelated patients documented worldwide.

**Methods:**

In this study, we investigated four patients from three families with ID. Whole-exome sequencing (WES) was performed to explore the genetic causes, while Sanger sequencing was used to confirm the identified variants. Furthermore, RNA sequencing and functional enrichment analysis were performed on patients with likely pathogenic variants to gain further insights into the molecular landscape associated with these variants.

**Results:**

Two novel variants in the *SHANK2* gene: a heterozygous splicing substitution (NM_012309.5:c.2198-1G>A p.Pro734Glyfs*22) in Family 1, and a heterozygous nonsense variant [NM_012309.5:c.2310dupT p.(Lys771*)] in Family 2 were identified by WES and confirmed by Sanger sequencing. RNA sequencing and cohort analysis identified a total of 1,196 genes exhibiting aberrant expression in three patients. Functional enrichment analysis revealed the involvement of these genes in protein binding and synaptic functions.

**Conclusion:**

We identified two novel loss of function variants that broadens the spectrum of *SHANK2* variants. Furthermore, this study enhances our understanding of the molecular mechanisms underlying *SHANK2*-related disorders.

## Introduction

Neurodevelopmental disorders (NDDs) are a group of mental health disorders resulting from the disruptions in crucial neurodevelopmental processes, leading to abnormal brain function that can affect emotions, cognition, learning, self-regulation, and memory (Morris-Rosendahl and Crocq, 2020). The severity and behavioral phenotypes observed in NDD patients vary widely, with diagnoses commonly including autism spectrum disorder (ASD), intellectual disability (ID), developmental delay (DD), and epilepsy (Zablotsky et al., 2019). Previous research has implicated various genetic variants in NDDs, including chromosomal rearrangements, copy number variants (CNVs), and coding-sequence variants. Although numerous genes have been associated with these disorders, each gene or genomic alteration typically accounts for less than 1% of cases. Many of the genes implicated in NDDs play a role in the development or functioning of neuronal circuits. Among the most extensively studied biological pathways in NDDs are those involving synaptic genes (Toro et al., 2010; Guilmatre et al., 2014; Hu et al., 2014; Leblond et al., 2014; Parenti et al., 2020).

The SH3 and multiple ankyrin repeat domains2 (*SHANK2*) gene is located on chromosome 11q13.3 and belongs to the *SHANK* gene family. *SHANK2* encode a pivotal scaffold protein in the postsynaptic density (PSD) complexes of glutamatergic synapses. The PSD is a specialized structure of the postsynaptic membrane that plays a critical role in neuronal signaling. The SHANK2 protein contains multiple domains facilitating protein–protein interactions and is vital for organizing the PSD through a complex network of molecular interactions (Sheng and Kim, 2000; Sasaki et al., 2020). In *Shank2* knock-out mice, both the ionotropic glutamate receptors at the synapse and the level of *Shank3* are upregulated. The mutant mice exhibit reduced dendritic spines and basal synaptic transmission. Moreover, they display remarkably hyperactive behavior and manifest significant autistic-like behavioral alterations, including repetitive grooming and deviations in vocal and social behaviors (Schmeisser et al., 2012; Won et al., 2012; Yoo et al., 2014).

Variants in the *SHANK2* gene have been implicated in individuals with ASD and ID. The initial discovery of *de novo* CNVs in the *SHANK2* gene in two unrelated patients was reported by Berkel et al. (2010), using microarray analysis. Subsequent investigations involved sequencing the *SHANK2* gene in a larger cohort of individuals, including 396 ASD cases, 184 cases of ID, and 659 unaffected individuals, leading to the identification of additional variants specific to ASD and ID (Berkel et al., 2010). In a study by Leblond et al., *SHANK2* was sequenced in 455 patients with ASD and 431 controls, and the findings were integrated with the previous research. A notable finding was the significant enrichment of variants affecting conserved amino acids in affected patients compared to controls. Furthermore, functional studies demonstrated a reduction in synaptic density at dendrites when neuronal cells were transfected with the variants identified in patients, as opposed to those exclusively detected in controls. These extensive investigations provide compelling evidence that certain *SHANK2* variants may confer an increased risk of ASD (Leblond et al., 2012).

Recently, there has been growing interest in utilizing total RNA sequencing in conjunction with whole-genome sequencing (WGS) or whole-exome sequencing (WES) to enhance our understanding of variant pathogenicity. This integrated approach enables the detection of outliers in both expressions and splicing, facilitating the interpretation of functional consequences (Kremer et al., 2017; Liu et al., 2022; Pan et al., 2022; Peymani et al., 2022). Moreover, it provides a valuable opportunity to investigate the molecular mechanisms underlying loss of function (LOF) variants in the *SHANK2* gene. In the present study, we investigated two novel *SHANK2* variants identified in three patient with ID from two families. Both the variants are LOF variants. Additionally, RNA sequencing and cohort analysis were performed on these patients to gain further insights into the impact of these LOF variants on gene expression. Through comprehensive analysis, we identified numerous genes with aberrant expression, which significantly contributed to our understanding of the molecular mechanisms associated with LOF variants in the *SHANK2* gene. These findings provide valuable insights into the pathogenicity of *SHANK2* variants and shed light on the underlying molecular processes involved in ID.

## Materials and methods

### Ethical compliance

Prior to their participation in this study, informed consent was obtained from all patients or their legal guardians. This research was conducted in accordance with ethical guidelines and regulations established by the ethics committee of the Second Affiliated Hospital of Chongqing Medical University (Approval No. 2022-549, dated 7 March 2022).

### DNA isolation, whole-exome sequencing, and variant analysis

Peripheral blood samples were collected from the patients using EDTA tubes, and genomic DNA was isolated using DNeasy Blood & Tissue kit (Qiagen, Hilden, Germany) according to the manufacturer’s instructions. A total of three microgram of genomic DNA was randomly fragmented and captured using the Agilent SureSelectXT V5 capture kit (Agilent Technologies, Santa Clare, CA). Sequencing was performed on an Illumina HiSeq2000 (Illumina, San Diego, CA) with 100-bp paired-end reads, following the recommended protocols. To ensure data quality, the raw sequencing reads underwent filtering using Fastp (Chen et al., 2018b) to obtain clean reads. FastQC was employed to evaluate the quality of the sequencing data in each sample (Trivedi et al., 2014). The clean DNA sequencing reads were aligned to the human reference genome hg19 (GRCh37) using the BWA-MEM algorithm (Li and Durbin, 2009). Ambiguously mapped reads (MAPQ < 10) and duplicated reads were removed using SAMtools (Li et al., 2009) and PicardTools,^1^ respectively. Single nucleotide polymorphisms (SNPs) and small insertions and deletions (INDELs) were identified following the best practices recommended by the Genome Analysis Toolkit software (McKenna et al., 2010). Variants were annotated using the Ensembl Variant Effect Predictor (McLaren et al., 2016). The ACGS Best Practice Guidelines for Variant Classification in Rare Disease 2020 were followed (Ellard et al., 2020). Classification of the variants into pathogenic (P), likely pathogenic (LP), benign (B), likely benign (LB), or variants of uncertain significance (VUS) was performed in accordance with the ACMG/AMP and ACGS guidelines (Richards et al., 2015; Ellard et al., 2020). All identified variants were further validated by Sanger sequencing.

### Minigene splicing assay

Functional analysis of the NM_012309.5:c.2198-1G>A variant was performed using an *in vitro* minigene assay. Genomic regions containing exons 18, 19, 20, 21, and intron sequences (2,355 bp) of *SHANK2* from proband 1 (individual II-1 from Family 1) were amplified using primers (SH2-F: 5′-tagtccagtgtggtggaattcATGTTGTCAAAGTCGGCCACA-3′, SH2-R: 5′-gccctctagactcgagcggccgcCCTTCTTCTTCCGGACCGAG-3′). The amplified fragments were then cloned into the pcDNA3.1(+) plasmid. Since proband 1 is heterozygous for this variant, clones of mutant-type hybrid minigene (minigene-MT) and wild-type hybrid minigene (minigene-WT) were obtained, respectively, and confirmed through Sanger sequencing. Subsequently, the hybrid minigenes were transduced into HEK293T cells with Lipofectamine 2000 reagent (Invitrogen). RNA was extracted 48 h after transfection followed by RT-PCR using the same upper primers. The resulting products were subjected to electrophoresis analysis and Sanger sequencing for further characterization.

### RNA isolation, sequencing and data preprocessing

Peripheral blood samples from the patients were collected using EDTA tubes. Subsequently, the red blood cells were removed through centrifugation after incubation with a red blood cell lysis solution. Total RNA sample was isolated within 24 h of collection and enriched using oligo-dT bead capture. Complementary DNA synthesis was performed following the manufacturer’s instructions, and libraries were prepared using the Illumina TrueSeq stranded mRNA sample prep kit (Illumina, San Diego, CA). Subsequently, sequencing of the pooled samples was conducted on a NovaSeq 6000 sequencing system. To obtain high-quality data, the raw sequencing reads underwent processing using Fastp to obtain clean reads (Chen et al., 2018a). Quality assessment of the sequencing data was performed using FastQC and mulitQC, evaluating factor such as sequence quality per base, sequence duplication level, and quality score distribution for each sample. The average quality score for the RNA sequences exceeded 30, indicating that substantial portion of high-quality sequences (Ewels et al., 2016). The clean RNA-sequencing reads were then aligned to the human reference genome (hg19) using STAR (2.4.2a) in conjunction with the Gencode v19 annotation (Dobin et al., 2013). Mapping evaluation metrics, including sequencing depth, percentage of mapped reads, and the number of expressed genes, were computed using DROP v1.21 (Yepez et al., 2021). Furthermore, the match between the RNA sequencing sample and its annotated DNA sample was assessed using DROP v1.21, with a cutoff of 0.8. Aberrant gene expressing was detected using DROP v1.21 (Yepez et al., 2021).

The clean RNA-sequencing reads were aligned to the human reference genome (hg19) using STAR (2.7.8a) along with the Gencode v29 annotation (Dobin et al., 2013). The Genomic Alignments R package’s “summarizeOverlaps” function was used for read counting. To enhance statistical power, we performed aberrant expression analysis by combining our data with 367 blood samples from GTEx data. Genes with a 95th percentile Fragments Per Kilobase of transcript per Million mapped reads (FPKM) less than 1 were considered as lowly expressed and were excluded from downstream analysis. OUTRIDER was employed to identify expression outliers (Brechtmann et al., 2018). Technical and biological covariates such as sex, age, and sequencing batch were automatically controlled by OUTRIDER, which utilized an autoencoder implementation. Genes were considered to have aberrant expression if they had an adjusted value of *p* < 0.05.

### Pathway enrichment analysis

To further explore the functional implications of the identified aberrations, we performed functional enrichment analysis using the KOBAS-I service (Bu et al., 2021). This comprehensive tool provides pathway enrichment analysis by leveraging various databases including GO, KEGG, Reactome, and GWAS catalogs. Pathways with an adjusted value of *p* < 0.05 were considered as significant, providing valuable insights into the biological relevance of the aberrant gene and their involvement in key pathways and biological processes.

## Results

### Clinical presentation

**Figure 1:**
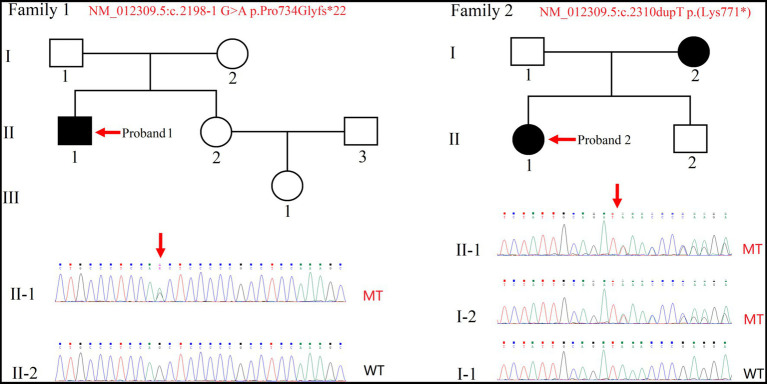
Pedigree of two families with intellectual disability. Sanger sequencing was performed on the probands (indicated by arrows). Squares and circles indicate males and females, respectively. Filled and empty symbols indicate affected and unaffected individuals, respectively. WT, wild-type; MT, mutant-type.

This study included three unrelated Chinese families (Figure 1; Supplementary Figure S1). Proband 1(individual II-1 from Family 1), a 27-year-old male, was the second child of non-consanguineous healthy parents. He had an uneventful full-term birth, walked at 3 years old, and began speaking at 7 years old. Proband 1 exhibited poor learning ability, limited mathematical skills, and discontinued education after the first grade of elementary school (Figure 1). Proband 2 (individual II-1 from Family 2), a 10-year-old female, was the first child of unrelated parents. The pregnancy and delivery were normal. The primary phenotype observed in this patient was mild ID. At the time of diagnosis, she was attending a regular primary school. Her brother was unaffected, but her mother had a diagnosis of mild ID (Figure 1).

Proband 3 (individual II-1 from Family 3) is a 4-year-old boy born to non-consanguineous healthy parents as their only child. He presented with globally development delay, ID and exhibited tendencies toward ASD features including impaired social interactions, repetitive behaviors, and delayed speech development. There was no reported family history of similar conditions or disorders (Supplementary Figure S1).

### WES analysis

Due to proband 1(individual II-1 from Family 1) being raised by grandparents while parents worked in another city, only singleton WES was performed, along with collection of peripheral blood from the proband’s sister. Through the analysis of WES data and variant pathogenicity classification following ACMG guidelines, only one variant in the *SHANK2* gene (NM_012309.5:c.2198-1G>A) were identified in the proband (Figure 1). Then, the variant was classified with criteria PVS1 + PM2 + PP3 and annotated as “LP” (Table 1). We have submitted this variant to ClinVar. It can be referenced under Submission Number: SUB13920791.^2^

**Table 1:** Variants identified in the *SHANK2* gene in probands in this study.

Proband	Localization	Nucleotide substitutions	AA change	SIFT	Polyphen2	CADD	SpliceAI	REVEL	Novel or ClinVar	Novel or HGMD	Population frequency [ExAC/gnomAD (non-neuro)]	Variant classification	ACMG classification
1	Intron 18	NM_012309.5:c. 2198-1G>A	p.Pro734Glyfs*22	N/A	N/A	33	Splice-Altering/strong	N/A	Novel	Novel	0/0	PVS1 + PM2 + PP3	LP
2	Exon 22	NM_012309.5:c.2310dupT	p.(Lys771*)	N/A	N/A	N/A	N/A	N/A	Novel	Novel	0/0	PVS1 + PP1_moderate+PM2	LP
3	Exon 3	NM_012309.5:c.178C>T	p.(Arg60Cys)	D	PD	24.4	N/A	Uncertain (0.36)	Novel	Novel	0.000104/0.0000385	PM2	VUS

Proband 1, individual II-1 from family 1. Proband 2, individual II-1 from family 2. Proband 3, individual II-1 from family 3. An asterisk (*) denotes variants resulting in premature termination codons. ExAC, Exome Aggregation Consortium projects; gnomAD (non-neuro), Genome Aggregation Database; REVEL, rare exome variant ensemble learner; N/A, not applicable; D, deleterious; PD, possibly damaging.

In Family 2, trio WES was conducted for proband 2 (individual II-1) and her parents, since the mother was also affected. This revealed only one variant in the *SHANK2* gene in the proband—NM_012309.5:c.2310dupT p.(Lys771*). Individual II-2, who is the younger brother of Proband 2, resides in a different region, and we were unable to obtain a sample from him (Figure 1). Then, the variant was classified with criteria PVS1 + PP1_moderate + PM2 and annotated as “LP” (Table 1). We have submitted this variant to ClinVar. It can be referenced under Submission Number: SUB13920833 (see footnote 2).

Then for proband 3 (individual II-1 from family 3), singleton WES was carried out due to cost considerations. Blood was collected to validate inheritance. Following ACMG guidelines, this analysis uncovered a variant in the *SHANK2* gene [NM_012309.5:c.178C>T p.(Arg60Cys)], which was classified based on criterion PM2 and annotated as “VUS” (Table 1). Another variant identified was in the *GLTSC1* gene [NM_015711.3:c.1324G>C p.(Ala442Pro)], also classified under criterion PM2 and labeled as “VUS.” Moreover, two variants were found in the *NBAS* gene [NM_015909.3:c.392C>T p.(Pro131Leu) and NM_015909.3:c.3674A>C p.(Glu1225Ala)]. Of these, the variant NM_015909.3:c.392C>T p.(Pro131Leu) was classified with criterion PM2 and annotated as “VUS,” while the variant NM_015909.3:c.3674A>C p.(Glu1225Ala) was classified using criteria PM2 + PP3 and also annotated as “VUS.” Diseases associated with *NBAS* predominantly affect the liver, which does not align with the ID phenotype observed in proband 3 (Staufner et al., 2020). Conversely, the *GLTSCR1* gene’s variants are implicated in the dominantly inherited NDD, Coffin-Siris syndrome-12 (Barish et al., 2020). Given our study’s emphasis on unveiling novel pathogenic variants in *SHANK2* and their potential mechanisms, our subsequent research will not encompass the less substantiated variant NM_012309.5:c.178C>T p.(Arg60Cys) identified in proband 3.

**Figure 2:**
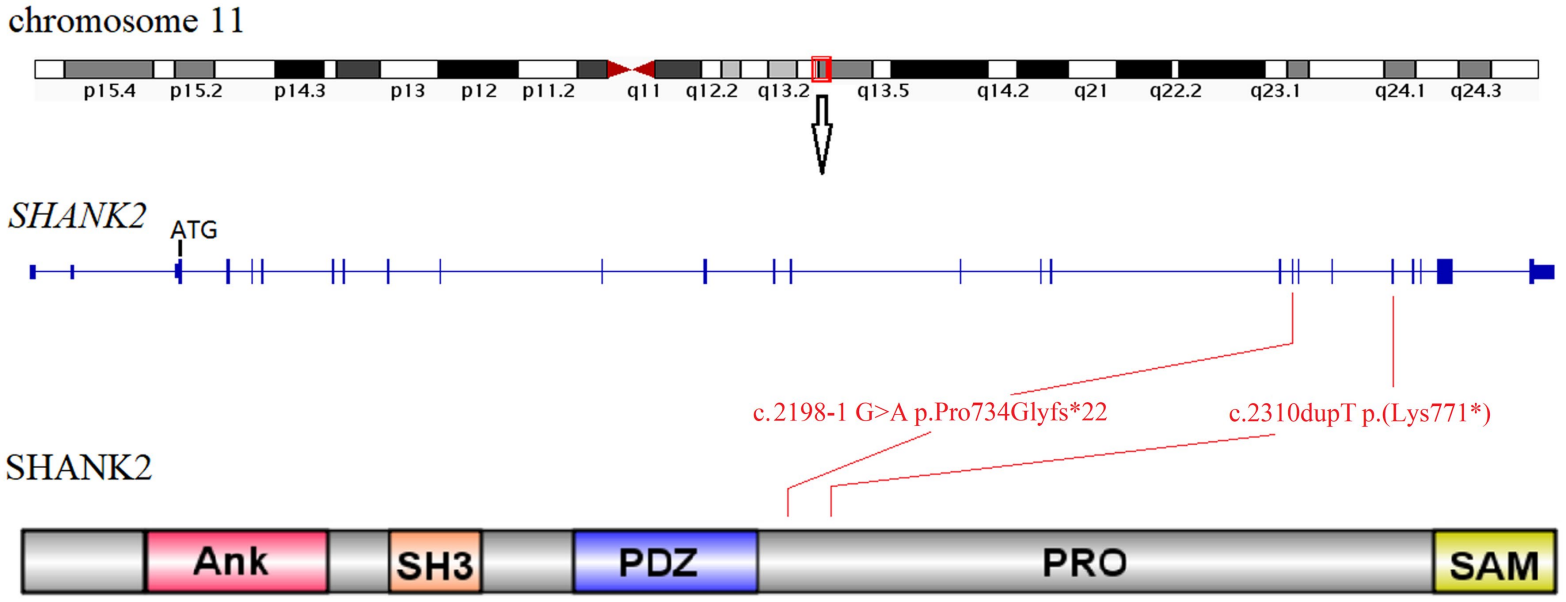
Location diagram of *SHANK2* variants identified in this study. The *SHANK2* gene is located on chromosome 11q13.3. The genomic structure of *SHANK2* is outlined in the middle diagram. The bottom cartoon shows the domains of human SHANK2 peptide. Variants identified in this study are mapped onto the gene and protein domains. Ank, ankyrin repeats; SH3, Src homology 3; PDZ, PSD95/DLG/ZO1; PRO, proline-rich region; SAM, sterile alpha motif.

Sanger sequencing was used to confirm the presence of these variants identified in the *SHANK2* gene (Figure 1; Supplementary Figure S1). The variants identified in proband 1 (individual II-1 from Family 1) and proband 2 (individual II-1 from Family 2) were novel. The NM_012309.5:c.2198-1G>A variant is a classical splice site variant. On the other hand, the NM_012309.5:c.2310dupT p.(Lys771*) variant immediately introduces a premature termination codon (TAA) due to the presence of a downstream AAA codon (Lys). Both of these LOF variants are located in the proline-rich region of the SHANK2 protein (Figure 2). Both LOF variants are classified as “LP,” while the NM_012309.5:c.178C>T p.(Arg60Cys) is interpreted as VUS based on the available evidence (Table 1).

### Minigene splicing assay

**Figure 3:**
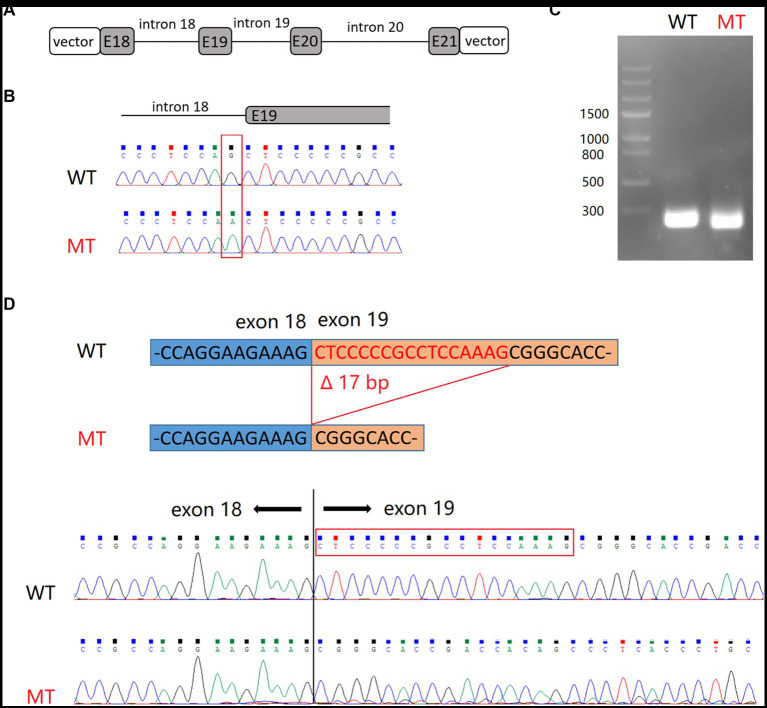
Minigene splicing assay for NM_012309.5:c.2198-1G>A variant in *SHANK2*. **(A)** Schematic representation of hybrid minigenes used in the assay. **(B)** The plasmids used in this assay were verified by Sanger sequencing. **(C)** Gel electrophoresis of RT-PCR products. **(D)** Sanger sequencing revealed that the product of minigene-MT exhibited skipping of the first 17 bp of exon 19. E, exon.

*SHANK2* is primarily expressed in the nervous system. However, due to ethical considerations, we were limited to obtaining peripheral blood samples from the patients. Given the low expression of *SHANK2* in peripheral blood, we resorted to the minigene assay to uncover the true impact of the NM_012309.5:c.2198-1G>A variant on pre-mRNA splicing. Subsequently, RT-PCR was employed to analyze the splicing products. Upon agarose gel electrophoresis, it was observed that cells transduced with minigene-WT produced a 240 bp band, whereas cells transduced with minigene-MT generated a 223 bp band. Subsequent Sanger sequencing verified that the minigene-WT product aligned with the reference sequence. Conversely, the minigene-MT product exhibited a skipping event of the first 17 bp of exon 19, leading to a frameshift and the generation of a premature stop codon (Figure 3).

### Transcriptome and cohort analysis

**Figure 4:**
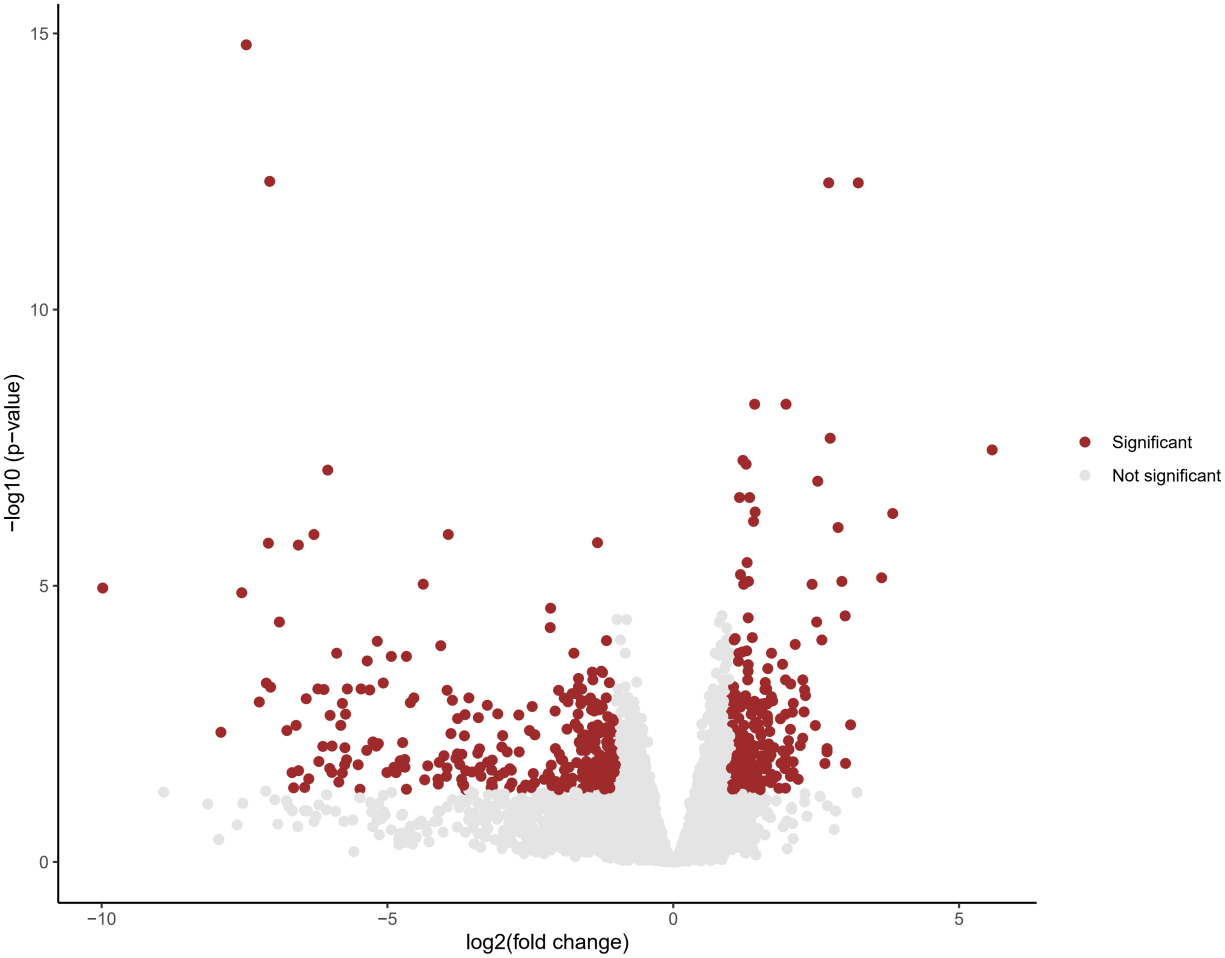
Volcano plot displaying differential gene expression in cohort analysis. Each data point represents a gene, plotted based on its fold change (log2) on the x-axis and the negative logarithm of the adjusted value of p on the y-axis. Genes with an adjusted value of *p* < 0.05 are considered statistically significant and are represented by red dots. Genes that do not reach statistical significance are shown in gray.

In order to investigate the potential molecular mechanism underlying these novel likely pathogenic variants, we performed RNA sequencing. Cohort analysis was conducted on RNA sequencing data from three patients of family 1 and 2, comparing them to publicly available databases as controls. A total of 1,196 genes were identified to exhibit aberrant expression patterns (Figure 4; Supplementary Table S1). Notably, among these genes, several were found to be associated with *SHANK2* and synapse function. One such gene is Glutamate receptor, ionotropic, N-methyl-D-aspartate associated protein 1 (*GRINA*). *GRINA* encodes a postsynaptic density protein involved in anchoring glutamate receptors (Schmeisser et al., 2012). Another gene of interest is *CTTN*, which encodes cortactin, an actin regulatory protein enriched at excitatory synapses (Mac Gillavry et al., 2016).

### Pathway enrichment analysis

**Figure 5:**
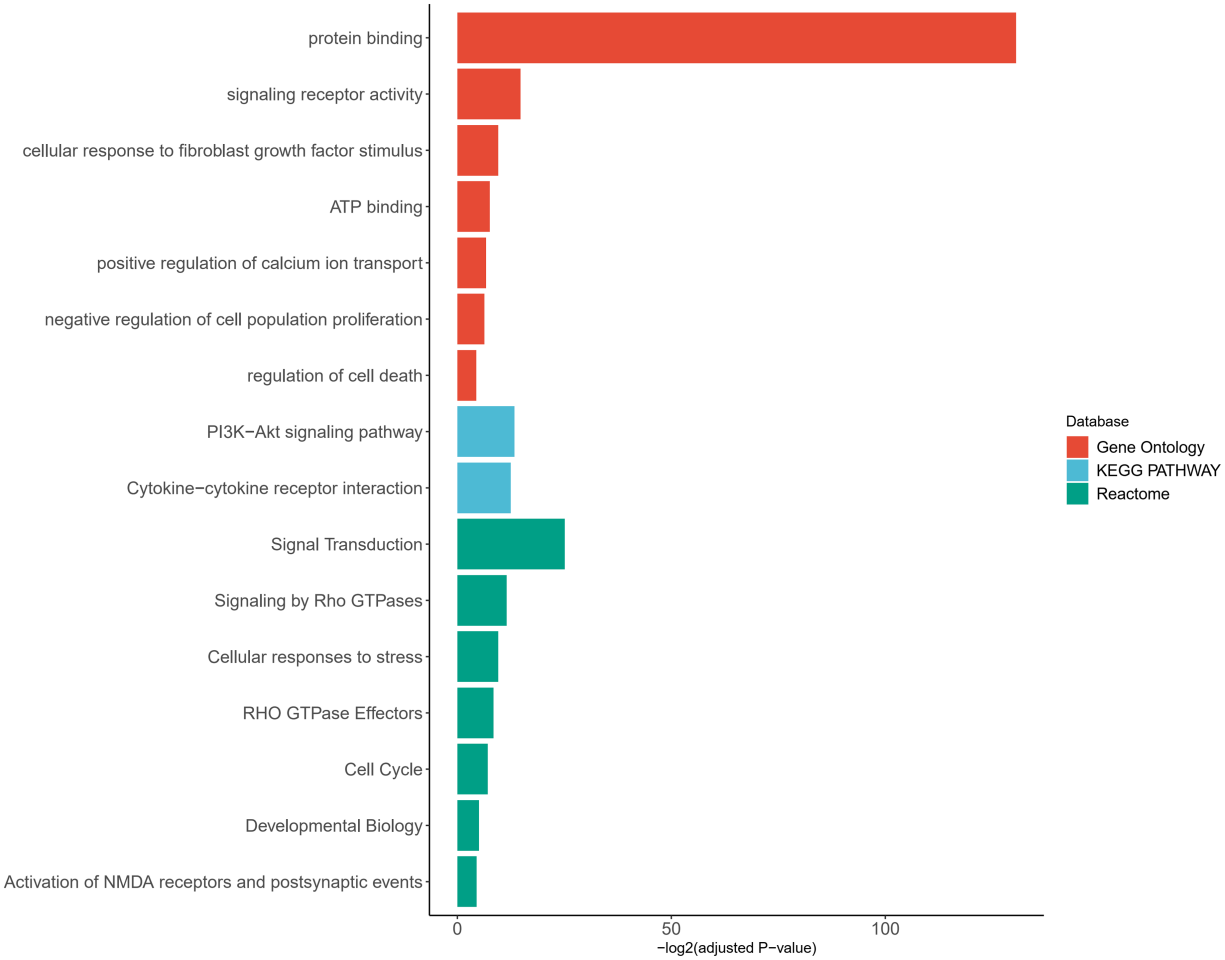
Pathway enrichment analysis of genes with aberrant expression. Pathways are categorized by different databases, represented by distinct colors. The X-axis represents the adjusted value of *p* with log transformation. Pathways that met the significance threshold (adjusted value of *p* < 0.05) were considered significant and included in the plot.

In order to further understand the functional implications of the aberrantly expressed genes, we performed pathway enrichment analysis. The functional annotation of these genes revealed their involvement in various biological pathways. Notably, a significant proportion of the genes were found to be associated with protein binding, indicating their participation in protein–protein interaction and molecular processes. Additionally, a subset of aberrantly expressed gene was found to be associated with the activation of NMDA receptors and postsynaptic events, further supporting their involvement in synaptic function and neuronal signaling. These findings are consistent with the known role of *SHANK2* as a postsynaptic scaffolding protein, highlighting the potential impact of the identified variants on synaptic organization and function (Figure 5; Supplementary Table S2).

## Discussion

The *SHANK* gene family, consisting of *SHANK1*, *SHANK2*, and *SHANK3*, encodes multi-domain master scaffold proteins that play critical roles in the organization and function of the postsynaptic density (PSD) complexes at glutamatergic synapses. SHANK proteins participate in various synaptic functions by interacting with many synaptic proteins (Guilmatre et al., 2014; Monteiro and Feng, 2017). Variants in *SHANK* genes have been repeatedly reported in individuals with a range of NDDs (Leblond et al., 2014; Doddato et al., 2022).

Among the *SHANK* gene family members, *SHANK2* is the largest gene and is located on chromosome 11q13.3 (Figure 2). Only 14 cases with *SHANK2* variants have been documented before. In this study, we present the identification of two novel *SHANK2* variants [NM_012309.5:c.2198-1G>A p.Pro734Glyfs*22 and NM_012309.5:c.2310dupT p.(Lys771*)] in two unrelated Chinese families. Both variants are located within the proline-rich region (PRO) of SHANK2 peptide. Out of the total 17 cases, seven individuals carried microdeletions encompassing *SHANK2* gene, while nine cases resulted in premature stop codons. Interestingly, the NM_012309.5:c.2198-1G>A p.Pro734Glyfs*22 variant reported in this study represents the first splicing variant identified in *SHANK2*, and is also considered a LOF variant (Table 2).

**Table 2:** Pathogenic variants identified in the *SHANK2* gene.

Patient index	cDNA change	AA change	Function	Phenotype	References
1	arr[GRCh37]11q13.3q13.4(70388251_70506824) × 1		LOF	ID, ASD	Berkel et al. (2010)
2	arr[GRCh37]11q13.4(chr11:70476560_70543234) × 1		LOF	ID, ASD	Berkel et al. (2010)
3	NM_012309.5: c.2521C>T	p.(Arg841*)	LOF	ID, ASD	Berkel et al. (2010)
4	arr[GRCh37]11q13.4(70476810_70542984) × 1		LOF	ID, ASD	Pinto et al. (2010)
5	arr[GRCh37]11q13.2q13.4(67768754_71286835) × 1		LOF	ID, ASD	Wischmeijer et al. (2011)
6	arr[GRCh37]11q13.3q13.4(70399609_70868619) × 1		LOF	ID, ASD	Leblond et al. (2012)
7	arr[GRCh37]11q13.3q13.4(70253389_72098888) × 1		LOF	ID, ASD	Leblond et al. (2014)
8	arr[GRCh37]11q13.2q13.4(67799160_71304541) × 1		LOF	ID	Marcou et al. (2017)
9	NM_012309.5: c.1896dupA	p.(Asp633Argfs*3)	LOF	ID	Bowling et al. (2017)
10	NM_133266.5: c.87C>G	p.(Tyr29*)	LOF	ID, ASD	Guo et al. (2018)
11	NM_133266.5: c.2540_2541del	p.(Ser847*)	LOF	ASD	Zhou et al. (2019)
12	NM_133266.5: c.1322delT	p.(Ile441Thsfs*8)	LOF	ID, ASD	Caumes et al., 2020
13	NM_133266.5: c.132dupA	p.(Asp45Argfs*3)	LOF	ID, ASD	Caumes et al. (2020)
14	NM_133266.5: c.334C>T	p.(Gln112*)	LOF	ID	Doddato et al. (2022)
15	NM_012309.5: c.2198-1G>A	p.Pro734Glyfs*22	LOF	ID	This study
16	NM_012309.5: c.2310dupT	p.(Lys771*)	LOF	ID	This study
17	NM_012309.5: c.2310dupT	p.(Lys771*)	LOF	ID	This study

Sixteen variants were collected from a total of 17 patients diagnosed with ID and/or ASD, including data from published literature and the current study. LOF, loss-of-function variants. An asterisk (*) denotes variants resulting in premature termination codons.

Through cohort analysis on the transcriptomic data from three patients carrying the identified novel LOF variants in *SHANK2*, a total of 1,196 genes exhibiting aberrant expression were identified. This dataset, derived from patient samples, presents a valuable resource providing insights into the molecular landscape associated with the disorder. *GRINA* belongs to the NMDA receptors (NMDARs) family. Studies conducted on mice lacking exons 6–7 of *Shank2* have demonstrated autistic-like behavioral abnormalities, which have been linked to altered N-methyl-Daspartate receptor (NMDAR) function. Furthermore, upregulation of *GRINA* has been consistently observed in various psychiatric diseases in human subjects (Schmeisser et al., 2012). Cortactin, encoded by the other noteworthy gene, is a monomeric protein located in the cytoplasm. Previous research in mice has revealed that Shank proteins play a crucial role in maintaining the stability of the spine actin cytoskeleton through their C-terminal cortactin binding site. Knockdown of *Shank* has been shown to significantly reduce the levels of cortactin in spines and increase the mobility of spine cortactin, as measure by single-molecule tracking photoactivated localization microscopy. These findings suggest that Shank proteins are involved in recruiting and stabilizing cortactin at the synapse, thus contributing to synaptic structure and function (Mac Gillavry et al., 2016). Our findings provides further evidence that *SHANK2* disruption can lead to molecular changes related to glutamate signaling and cytoskeletal dynamics, which may contribute to the neurodevelopmental phenotypes. However, it is important to acknowledge that expanding the sample size by including more patients would be highly beneficial. This approach would lead to a more comprehensive understanding of the spectrum of gene expression abnormalities related to this disorder. Additionally, it would also facilitate the identification of additional *SHANK2* variants.

While NDDs caused by *SHANK2* variants exhibit autosomal dominant inheritance, the severity and specific behavioral phenotypes observed in individuals display a high degree of variability, including possible incomplete penetrance. In our clinical cohort, proband 3 (individual II-1 from family 3) who carried a NM_012309.5:c.178C>T p.(Arg60Cys) variant in *SHANK2* (Table 1; Supplementary Figure S1). Interestingly, this specific variant in *SHANK2* corresponds to the R12C alteration in the *SHANK3* SPN domain, which has been previously implicated as a potential pathogenic variant in AD patients (Leblond et al., 2014; Sasaki et al., 2020). However, it is important to note that this variant we discovered was inherited from his father, who exhibits no clinical phenotype. The combined Annotation Dependent Depletion (CADD) score is 24.4 and multiple in-silico programs consistently predicted the deleterious effect (Table 1). However, its REVEL score is only 0.36, which categorizes it as “Uncertain” (Ioannidis et al., 2016). Therefore, this variant was classified as VUS according to the ACMG criteria (Table 1; Supplementary Figure S1).

This study represents a preliminary investigation into the transcriptional changes associated with *SHANK2* variants and NDDs. A clear limitation is that the ideal neural tissues were not examined due to clinical inaccessibility and ethical considerations. Instead, we analyzed the more readily accessible peripheral blood nucleated cells, hoping to uncover valuable insights. Pathway enrichment analysis of the differentially expressed genes did reveal associations with synaptic function and neuronal signaling. While we cannot explain the underlying mechanisms for these findings, we speculate that they may be related to mechanisms such as neuroendocrine signaling, albeit without direct evidence. Additionally, our study is limited by the small cohort size, comprising only three patients harboring two LOF variants. Moreover, patient II-1 from family 2 in this study inherited the variant from his affected mother. Considering the substantial phenotypic variability associated with such disorders, the inclusion of only three patients in our study is insufficient to draw definitive conclusions. Therefore further expansion of the patient cohort is warranted to obtain more robust and comprehensive data.

In summary, our study contributes to the current understanding of the genetics and clinical manifestations associated with *SHANK2* variants. Through the integration of WES and RNA-seq analyses, we have gained significant insights into the pathogenicity of two novel *SHANK2* variants identified in three patients diagnosed with ID. These findings enhance our understanding of the molecular mechanisms underlying *SHANK2*-related disorders and have implications for clinical diagnosis and management in affected individuals.

## Data availability statement

The raw sequence data reported in this study have been deposited in the Genome Sequence Archive (Chen et al., 2021) in National Genomics Data Center (CNCB-NGDC Members and Partners, 2022), China National Center for Bioinformation/Beijing Institute of Genomics, Chinese Academy of Sciences (GSA-Human: HRA005493) that are publicly accessible at https://ngdc.cncb.ac.cn/gsa-human.

## Ethics statement

The studies involving humans were approved by the Ethics Committee of the Second Affiliated Hospital of Chongqing Medical University. The studies were conducted in accordance with the local legislation and institutional requirements. Written informed consent for participation in this study was provided by the participants’ legal guardians/next of kin.

## Author contributions

YW: Funding acquisition, Investigation, Methodology, Writing – original draft. WL: Investigation, Methodology, Writing – original draft. BT: Conceptualization, Resources, Software, Supervision, Writing – review & editing. SL: Conceptualization, Supervision, Writing – review & editing.

## Footnotes

^1^ http://broadinstitute.github.io/picard/

^2^ https://ncbi.nlm.nih.gov/clinvar/
