# Supplementary material for: Identification of novel SHANK2 variants in two Chinese families via exome and RNA sequencing
Source: Front Neurosci. 2023 Nov 24;17:1275421. doi: 10.3389/fnins.2023.1275421 (PMC10704150; doi:10.3389/fnins.2023.1275421)
Supplement: Supplementary file 2 [file Data_Sheet_1.docx]

Supplementary Material

# Supplementary Figures

##
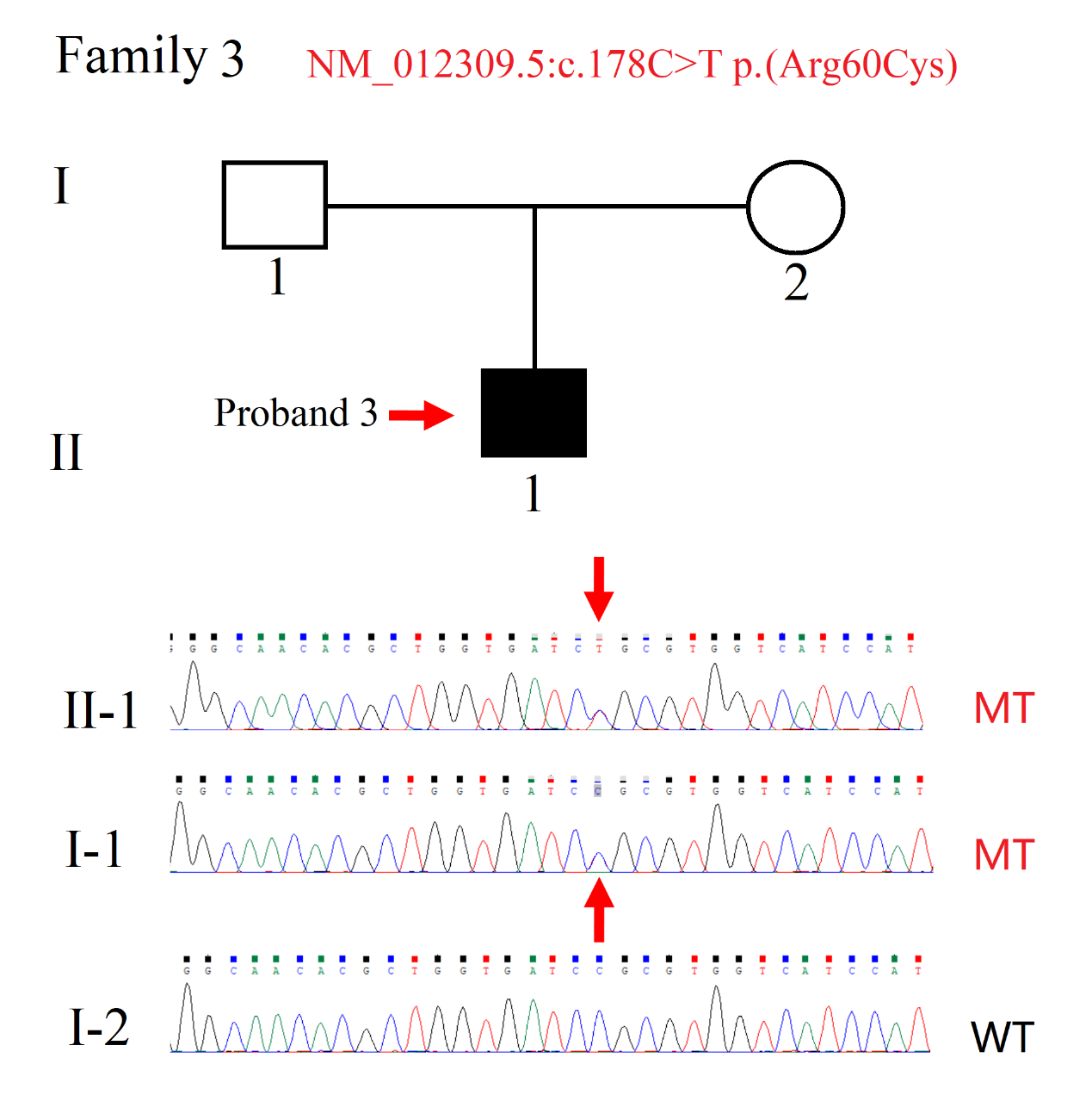


**Supplementary Figure 1:** Pedigree of family 3 with intellectual disability. Sanger sequencing was performed on the proband and his parents. Squares and circles indicate males and females, respectively. Filled and empty symbols indicate affected and unaffected individuals, respectively. The heterozygous peak for the proband’s father (individual I-1)) is subtle; careful inspection of the sequence trace is needed to distinguish the mixed peaks indicating heterozygosity.
